# Supplementary material for: Investigation of new inflammatory biomarkers in patients with brucella
Source: PLoS One. 2024 Feb 15;19(2):e0297550. doi: 10.1371/journal.pone.0297550 (PMC10868832; doi:10.1371/journal.pone.0297550)
Supplement: Attachment — Submitted filename: respond to reviewer plos one.doc [file pone.0297550.s001.doc]

**Response to reviewer**

We have corrected our article in accordance with reviewer recommendations.

Journal Requirements:

1. Please ensure that your manuscript meets PLOS ONE's style requirements, including those for file naming

**Response:** Our manuscript meets PLOS ONE's style requirements, including those for file naming.

2. We suggest you thoroughly copyedit your manuscript for language usage, spelling, and grammar.

**Response:** We corrected us thoroughly copyedit our manuscript for language usage, spelling, and grammar.

3. We noticed you have some minor occurrence of overlapping text with the following previous publication(s), which needs to be addressed:

https://onlinelibrary.wiley.com/doi/10.1111/ijd.12959

https://cyberleninka.org/article/n/985393

https://ijponline.biomedcentral.com/articles/10.1186/s13052-014-0095-1

<https://dergipark.org.tr/en/pub/cbusbed/article/887818>

**Response:** We are corrected some minor occurrence of overlapping text with the some publication. We are co-authors on some of these publications. The similarities in the articles are due to the same ELISA method being used.

4. Thank you for stating the following financial disclosure: "This publication was supported by Dicle University Scientific Research Projects Commission, dated 11.08.2021, with project number TIP.20.037." Please state what role the funders took in the study. If the funders had no role, please state: ""The funders had no role in study design, data collection and analysis, decision to publish, or preparation of the manuscript."" If this statement is not correct you must amend it as needed. Please include this amended Role of Funder statement in your cover letter; we will change the online submission form on your behalf.

**Response:** We added this sentence ‘The funders had no role in study design, data collection and analysis, decision to publish, or preparation of the manuscript’

5. Your ethics statement should only appear in the Methods section of your manuscript. If your ethics statement is written in any section besides the Methods, please move it to the Methods section and delete it from any other section. Please ensure that your ethics statement is included in your manuscript, as the ethics statement entered into the online submission form will not be published alongside your manuscript.

**Response:** We have included our ethical statement in the Methods section of our article. We deleted ethical statement in the discussion section of our article.

6. Please review your reference list to ensure that it is complete and correct.

**Response:** We reviewed our reference list to ensure that it is complete and correct

Reviewer 1:

1. Abstract: It would be appropriate to write the full value of p instead of the p value of iris "<0.05" in the results in the abstract.

**Response:** We changed p value of the irisin as ‘p=0.045’

2. Materials and methods: What are the diseases or conditions in which the levels of the studied biomarkers are affected? Were these taken into consideration in the patients included in the study?

**Response:** It is known that different diseases such as gastrointestinal or nutritional disorders, metabolic diseases can significantly affect the levels of irisin, presepsin and apelin. Therefore, persons with any disease including respiratory, endocrinology, cardiac, metabolic or renal diseases and those with viral hepatitis were not included in the study.

We added this sentence ‘Patients with respiratory, endocrine, cardiac, metabolic, renal or hepatic disease and pregnant women were also excluded’ in the Methods section.

3. Results: It is recommended that the p value of irisin be written exactly like that of other biomarkers in Table 1. The exact p values of other biomarkers are written, but irisin is written as p < 0.05.

**Response:** We changed p value of the irisin as ‘p=0.045’ in the Table 1.

4. Discussion: What could be the reasons why presepsin was not statistically significant in your study? It is recommended that you write your reasons in the discussion section. It seems that some of the studies conducted with presepsin were not significant due to reasons such as the patients' comorbidities not being standardized. Additionally, the number of patients included in the study may also change the significance.

**Response:** We had written some sentences in the discussion section for this reason.

And we added this sentence ‘This result has been attributed to the slower course of the inflammatory response in the brucellosis as compared to other diseases and the limited number of patients in our study’ in the discussion section.

Reviewer 2:

1. Exclusion criteria to be included in the study; Co-infections/ Co-morbidity cases are not mentioned anywhere in the article.

**Response:** It is known that different diseases such as gastrointestinal or nutritional disorders, metabolic diseases can significantly affect the levels of irisin, presepsin and apelin. Therefore, persons with any disease including respiratory, endocrinology, cardiac, metabolic or renal diseases and those with viral hepatitis were not included in the study.

We added this sentence ‘Patients with respiratory, endocrine, cardiac, metabolic, renal, hepatic disease and pregnant women were also excluded’ in the Methods section.

2. Control selection is partially met. Along with healthy controls, other febrile illness groups could have been enrolled. This gives appropriate conclusions for the results.

**Response:** This study was designed for patients with Brucella. In the future, we plan to conduct this study with a larger number of patients and more different patient groups.
